# Intercellular junction-driven stromal cell stacking in a confined 3D microcavity

**DOI:** 10.1063/5.0197187

**Published:** 2024-11-08

**Authors:** Avelino Dos Santos Da Costa, Hyuntae Jeong, Ramesh Subbiah, Kwideok Park, In-Suk Choi, Jennifer H. Shin

**Affiliations:** 1 Department of Materials Science and Engineering, Seoul National University, Seoul 08826, Republic of Korea; 2 Center for Biomaterials, Korea Institute of Science and Technology (KIST), Seoul 02792, Republic of Korea; 3 Department of Mechanical Engineering, Korea Advanced Institute of Science and Technology (KAIST), 291 Daehak-ro, Yuseong-gu, Daejeon 34141, Republic of Korea; 4 Division of Biomaterials and Biomechanics, Department of Restorative Dentistry, School of Dentistry, Oregon Health and Science University (OHSU), Portland, Oregon 97201, USA; 5 Division of Bio-Medical Science and Technology, KIST School, University of Science and Technology (UST), Seoul 02792, Republic of Korea

## Abstract

Understanding the detailed mechanisms driving fibroblast migration within native tissue settings during pathophysiological events presents a critical research challenge. In this study, we elucidate how stromal cells migrate and contribute to the development of three-dimensional (3D) cellular aggregates within confined microcavities. Integrin α5β1 and β-catenin (β-cat) are central in guiding this collective migration and achieving optimal filling of the microcavity. When β-cat is suppressed, cells tend to migrate more sporadically, leading to less efficient cellular organization. Furthermore, we also detail the pivotal roles of Cx43 and N-cadherin (N-cad) in orchestrating collective migration and in shaping efficient cellular stacking. Suppressing gap junctions, especially Cx43, significantly impacts the extracellular matrix expression, integrin α5 and β1, and other elements in the 3D construct, emphasizing the importance of physicochemical cell–cell interactions. The distribution patterns of N-cad and focal adhesion kinase (FAK) further corroborate the essential roles in forming cell–cell junctions and FAK in establishing the foundational layer that underpins the cell stacking within the microcavity. Interestingly, neither Rho-associated protein kinase (ROCK) nor RhoA significantly alter the cell migration pattern toward microcavity. These findings provide fresh perspectives on fibroblast activities in 3D space, enriching our understanding and offering implications for advancements in wound healing and tissue engineering.

## INTRODUCTION

Wound healing is a complex process involving numerous cell types such as epithelial cells, endothelial cells, keratinocytes, fibroblasts, dermal, mesenchymal tissues, blood vessels, nerves, and immune cells.^1,2^ One critical component of this process is the formation of granulation tissue, where stromal cells are recruited to the wound bed through the secretion of growth factors such as transforming growth factor (TGF)-β1, fibroblast growth factor (FGF), and platelet-derived growth factor (PDGF) by macrophages.^3–5^ As this occurs, migrating fibroblasts also secrete extracellular matrix (ECM) necessary for epithelial cell migration and tissue restoration.^6,7^ This results in the creation of a three-dimensional (3D) fibroblast-ECM structure at the wound site, essential for proper tissue healing. The exact mechanisms directing the collective migration of stromal cells within these 3D tissue settings, however, remain elusive.^8,9^ While *in vitro* models, such as organoids^10^ or spheroids,^11–13^ have been used to simulate 3D arrangements of certain cell types, however, the collective migration of stromal cells and the mechanisms governing their movement within 3D tissue environments are not fully understood.^8,9^ The migration of stromal cells, particularly fibroblasts, is primarily influenced by cell-substrate adhesions, integrins, and the formation of focal adhesions.^14–16^ These adhesion molecules enable fibroblasts to attach to the ECM and regulate their migratory behavior.^17–19^ Unlike closely connected networks of epithelial or endothelial cells,^20,21^ fibroblasts rely less on cell–cell adhesions with transient cell–cell junction^22^ and primarily move through cell–ECM interactions within their tissue microenvironment.^23,24^ However, in pathophysiological conditions, fibroblasts can form clusters or aggregates within or around ECM proteins.^25,26^ In such situations, fibroblasts undergo phenotypic changes, such as transitioning to myofibroblasts, which are critical for wound healing, tissue repair, and fibrosis. Activated fibroblasts synthesize and deposit ECM components, leading to the formation of a dense fibrous network. This altered behavior can result in the formation of cell aggregates, particularly in response to tissue injury or inflammation, where fibroblasts collectively work to facilitate repair processes. During this collective behavior, cell–cell adhesions and gap junctions play essential roles in coordinating cell movement and communication, ensuring effective tissue repair and remodeling.^27–29^ Previous studies have demonstrated that fibroblasts exert force on fibronectin to close gaps and that matrix assembly focal adhesion kinase (FAK) kinase activity is crucial for this process.^30^ Additionally, our previous study confirmed that stromal cells close voids using the purse string contraction mechanism, a well-known mechanism employed by epithelial cells on 2D substrates.^31^ While there has been extensive exploration of individual cell migration in 3D microenvironments, resulting in various migration types such as ameboid, mesenchymal, or lobopodial,^32^ collective migration is a prevalent phenomenon in physiological processes such as embryonic development,^33^ wound healing,^34^ immune responses,^35^ and cancer cell migration.^36^ Therefore, understanding the migration mechanisms of cells in a 3D microenvironment is crucial for mimicking physiological and pathological processes.

In this study, we present a microcavity with curved topography, fabricated from a polydimethylsiloxane (PDMS)-based material. This substrate consists of microcavity that ranges from 100 to 400 *μ*m for its side length (L_s_), and a thickness of approximately 110 *μ*m (Fig. S1). Prior to cell culture, the PDMS surface was coated with a 2 mg/mL solution of polydopamine (PDA) for higher cell attachment. Contrary to the previously understood fibroblast migration mechanism, we show that cells migrate collectively toward the microcavity, aiding in the formation of structures that recapitulate granulation tissue formation.

## RESULTS AND DISCUSSION

### Stacked layers of stromal cells

**FIG. 1.**
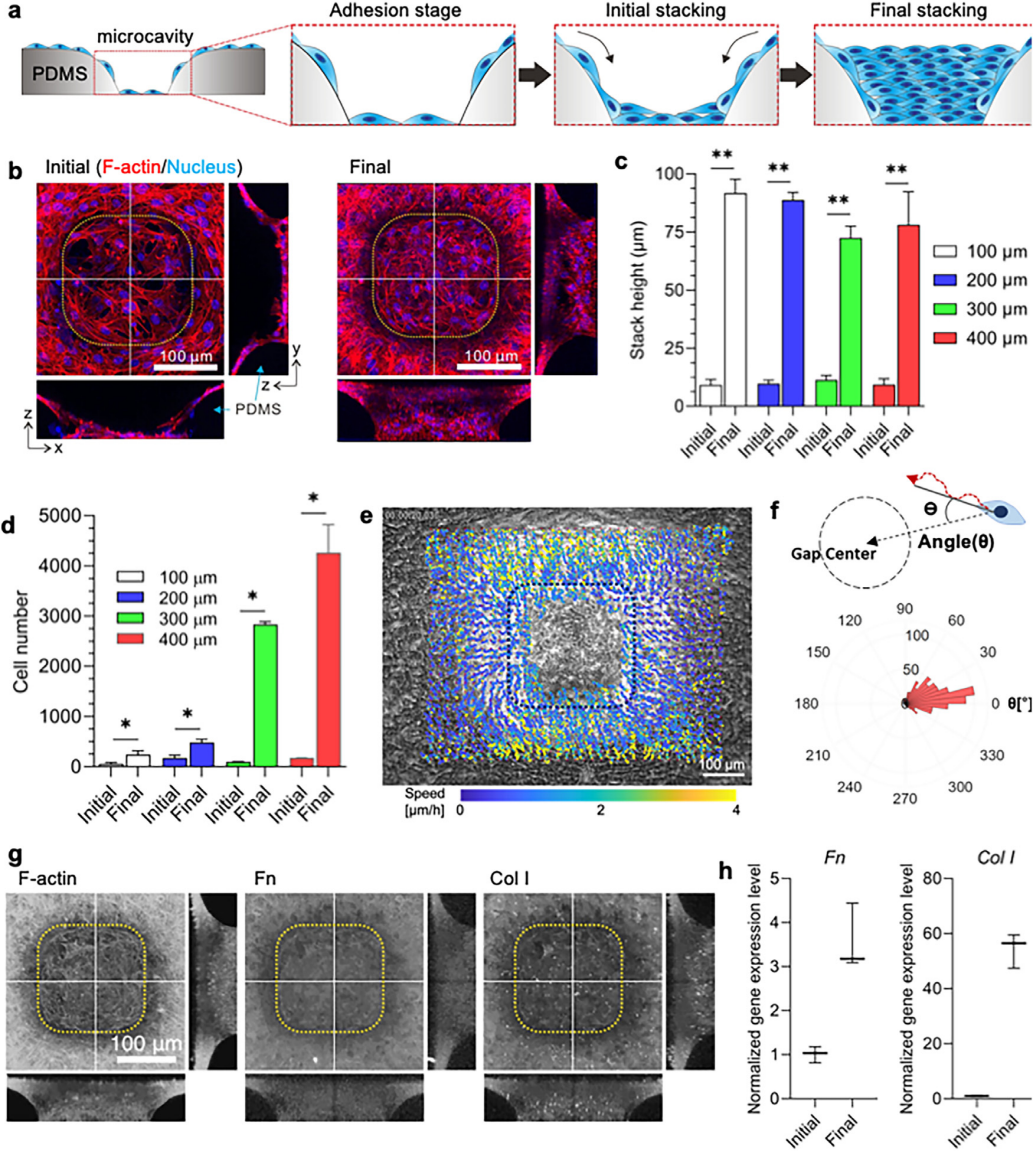
NIH 3T3 cells stacking on 3D microcavity. (a) The illustration shows the stages of stacking formation in NIH 3T3 mouse fibroblast cells. The cells crawl down from the reservoir, as indicated by the arrows. After 72 h, the stacking is fully formed within the microcavity. (b) 3D reconstructions of confocal images, displaying the z-projection and corresponding y–z and x–z sections of cells (along the white lines). The cells are stained for F-actin (red) and the nucleus (blue) at the initial (left) and final (final) stages of stacking cell formation. The yellow dotted boxes indicate the boundaries of the PDMS substrate. The scale bars represent 100 *μ*m. (c) The plot shows the height of the initial and final stages of stacking cell formation for different gaps ranging from L_s_ = 100–400 *μ*m. The results demonstrate that the final stage of stacking cells has a significantly greater cell height compared to the initial stage. Statistical analysis was performed using Kolmogorov–Smirnov test, comparing the initial cell height of each group as the control, ^**^P < 0.01, *n =* 5, error bars show standard deviation. (d) The plot presents the number of cells at the initial and final stages of stacking formation for various microcavity side lengths. Statistical analysis was conducted using the Kolmogorov–Smirnov test, comparing the cell number at the initial stage as the control, ^*^P < 0.05, *n =* 4, error bars show standard deviation. (e) Region of interest (ROI) for migration trajectory analysis around the microcavity represented by L_s_ = 100 *μ*m. The scale bar is 100 *μ*m. (f) Determination of the angle during cell migration from the reservoir to the microcavity. The rose plot represents the migration direction from the initial point to the final point during the stacking formation from (e). (g) This is a 3D reconstruction of confocal images stained for F-actin, Fn, and Col I at the final stage of stacking formation along the white lines represented by L_s_ = 100 *μ*m. Single-channel micrographs have been presented in grayscale to improve contrast and clarity. Scale bar: 100 *μ*m. h, These plots display gene expression of Col I and Fn with GAPDH as the housekeeping gene, indicating the upregulation of both genes during the final stage of stacking cell formation. Statistical analysis was performed using Wilcoxon test, comparing D1 (initial time) as the control, *n =* 3, error bars show standard deviation.

The NIH 3T3 mouse fibroblast cells were seeded on the substrate and allowed to migrate into the microcavity over a 3-day period [Fig. 1(a)]. Initial observations at 24 h after cell seeding revealed the formation of a monolayer within the microcavity [Fig. 1(b), left, and Movie S1]. By 72 h, the cells developed a complete stacking formation with multiple layers filling the microcavity completely [Fig. 1(b), right and Movies S2 and S3]. Further analyses revealed that this stacked cell formation occurred not only in microcavities of 100 *μ*m L_s_ (L_s_ =100 *μ*m) but also in larger ones up to L_s_ = 400 *μ*m (Fig. S2 and Movies S4–S6). Upon completion of the experiment, a tenfold increased stacking of the cells was observed compared to the initial layers of cells across all microcavity side lengths [Fig. 1(c)]. The increased stacking of the cells resulted in a significantly larger cell population at the final time point compared to the initial time point [Fig. 1(d)]. Although the starting cell population was similar across all samples, the cell count increased proportionally to the microcavity area over time. These data suggest that the cellular stack formation can be attributed not only to inherent cell proliferation but also to the collective migration of cells toward the microcavity from the reservoir, underscoring the crucial role of migrating cells in 3D structure formation.

To verify the source of cell recruitment into the confined microcavity, we utilized particle image velocimetry (PIV) based on time-lapse microscopy data [Fig. 1(e)]. By tracing the collective migration of cells from the reservoir toward the microcavity, the direction of velocity and trajectory vectors were analyzed [Fig. 1(f) and Movie S7]. Data showed a prevalent cell movement direction toward the microcavity (0° representing movement toward the microcavity). This finding confirms the persistence of collective cell migration from the reservoir toward the microcavity, thus supporting the recruitment of cells into the confined microcavity from the reservoir. In summary, our results demonstrate that the stacking fibroblasts are collectively recruited from the reservoir of the substrate, fill the microcavity, and subsequently form a fully stacked 3D structure of stromal fibroblasts. Furthermore, to investigate whether stacking cell formation is limited to NIH 3T3 mouse fibroblast cells, we conducted experiments using human dermal fibroblast cells (hDFB). The hDFB cells too exhibited stacking after 72 h (Fig. S3, refer to statistical analysis in the Method section). However, with epithelial HaCaT cells, the formation of cellular stacking did not occur, even when the microcavity side length was reduced to L_s_ = 50 *μ*m [Figs. S4(a)–S4(d) and S4(f), refer to statistical analysis in the Method section and Movies S8 and S9]. While the number of cells in the final stage increased compared to the initial stage due to the dense nature of HaCaT cells, the total number was significantly lower than that of hDFB cells, also demonstrating the non-stacking behavior of epithelial cells [Figs. S4(e) and S4(g), refer to statistical analysis in the Method section]. This pattern suggests that stacked cell formations are a characteristic behavior of stromal cells, not epithelial cells. Notably, stromal cells formed stacking structures in the microcavity, while HaCaT cells representing epithelial cells were unable to do so. We attribute this difference to the curved surface topography of the microcavity, which requires higher tension exerted from the cells as well as the ability of the cells in secreting ECM during crawling toward the confined space.

Stromal cells, under natural conditions, are responsible for ECM secretion during wound healing processes.^6^ To discern a possible link between ECM secretion and stacked cell formation, we checked for ECM markers, specifically fibronectin (Fn) and collagen I (Col I), through immunofluorescence staining. We also confirmed gene expression level and to facilitate this analysis, we used a separate microcavity substrate that fits into a 6-well tissue culture plate for real-time PCR and western blot analysis (Fig. S5, refer to statistical analysis in the Method section). The results revealed abundant presence of Fn and Col I in the final stacking cell formation [Fig. 1(g)]. Additionally, real-time PCR analysis confirmed a significant upregulation of Fn and Col I gene expression at the final stage [Fig. 1(h), refer to statistical analysis in the Method section]. These findings indicate persistent secretion of ECM components over time until the final stage of cell stacking, which is day 3, exhibited a heightened level of the ECM components, including as Fn and Col I. This implies that these stacking cells might emulate the properties of granulation tissue, known for its ECM-rich composition.^37^ Overall, our investigation establishes that the origin of the stacked cells in a microcavity is the reservoir, and there is a continuous secretion of ECM components throughout their formation.

### The efficacy of stacking depends on the collective migration of cells toward microcavity

**FIG. 2.**
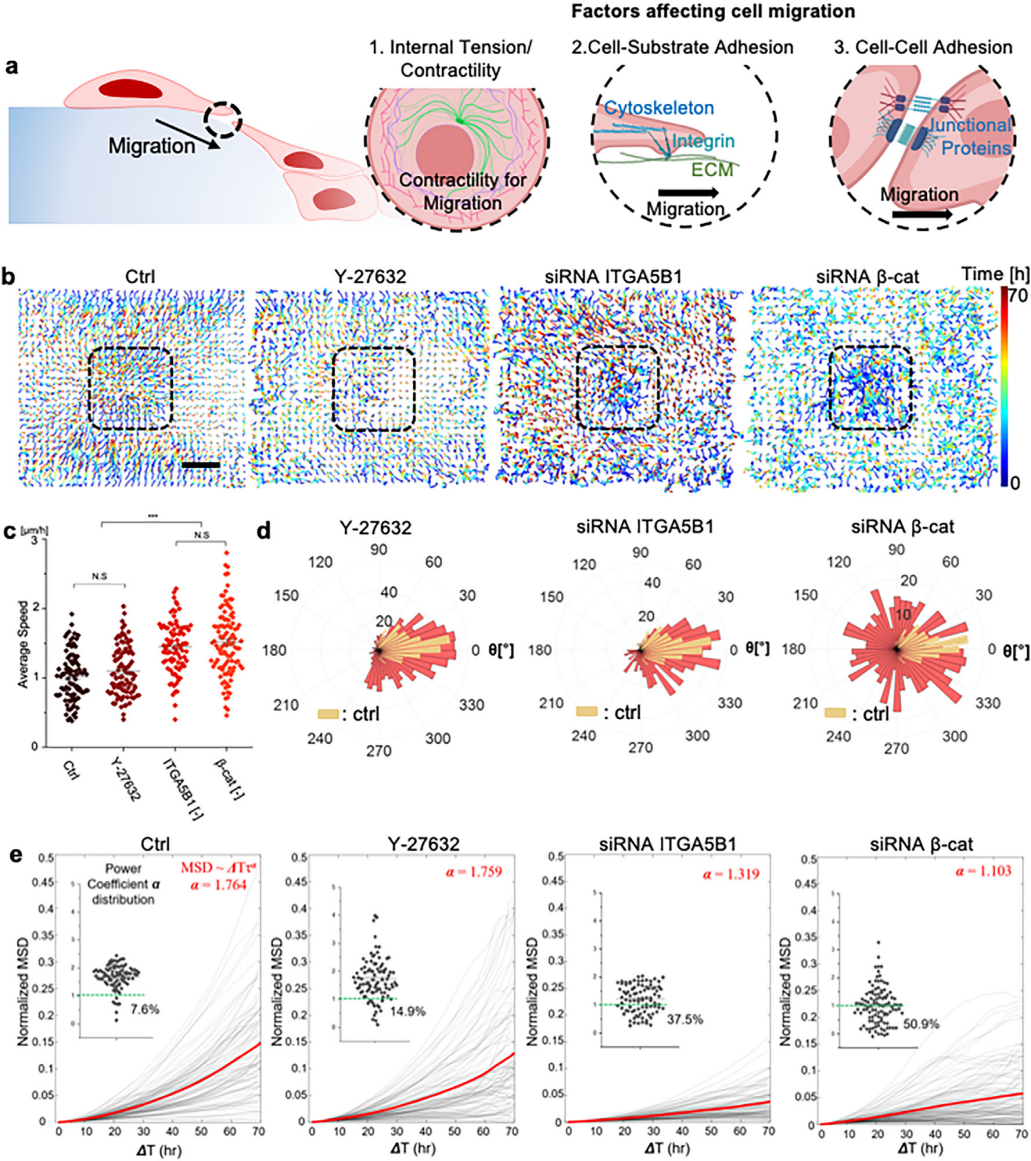
Collective migration behaviors in mediating stacking cell formation. (a) This illustration depicts the proposed mechanisms for the cell crawling down process, involving contractility, cell-substrate adhesion (integrin), and cell–cell junctions. (b) ROI for migration trajectory analysis around the microcavity, comparing control, Y-27632 treatment, siRNA ITGA5B1 transfection, and siRNA β-cat transfection groups. The scale bar is 100 *μ*m. (c) Plot of the velocity for each group. Statistical analysis was conducted by using the Kruskal–Wallis test (SPSS) comparing the non-treatment group as the control, ^***^P < 0.001, data points represent each individual cell. (d) Rose plots along the migration path from the initial point to the final point during the stacking formation for each group. These plots indicate persistent migration for siRNA ITGA5B1 transfection and Y-27632 treatment, while dispersed migration is observed for siRNA β-cat transfection relative to the microcavity's direction. (e) Mean square displacement (MSD) of the cells according to the progress of time (ΔT) in each group, showing higher MSD during Y-27632 treatment compared to the other groups.

To conduct a comprehensive assessment of the efficacy of cell stacking, we analyzed the directional migration of cells toward the microcavity. Our objective was to determine the trajectories and velocities of collective cell migration across different experimental groups, such as control (untreated), Y-27632 treatment, siRNA ITGA5B1 transfection, and siRNA β-cat transfection. Our hypothesis suggests that the effective formation of stacking cells occurs through migration along the PDMS surface toward the microcavity that is facilitated by cell-substrate adhesion through integrin and cell–cell adhesion [Fig. 2(a)]. To validate this hypothesis, we employed particle image velocimetry (PIV) based on time-lapse microscope images.

Initially, we evaluated the effectiveness of the siRNAs by culturing cells on a separate tissue culture plate and treating them with siRNA targeting integrin α5β1 (ITGA5B1) and β-cat. The results confirmed a marked downregulation of both integrin α5 and integrin β1 gene expression levels, as confirmed by real-time PCR and western blot analyses [Figs. S6(a)–S6(d), refer to statistical analysis in the Method section]. Additionally, siRNA β-cat efficacy was confirmed through the detection of decreased β-cat gene expression level using real-time PCR [Fig. S6(e)]. Once confirmed, we transfected the siRNA-treated cells onto a microcavity substrate to evaluate their impact on stacking cell formation. We tracked the collective cell migration from the reservoir to the microcavity in both temporal and spatial dimensions using L_s_ = 100 μm as the representative microcavity [Fig. 2(b)]. To track the temporal difference in cell migration, we utilized color-coded trajectories correlating with time by accumulating the PIV-calculated velocity fields (Fig. S7). Most cell trajectories displayed synchronized directionality of the migration at subsequent time points, with the exception of the siRNA β-cat condition. The component analysis of cell velocity also confirmed the predominance of the velocity component directed toward the center point over the perpendicular velocity component, with the exception of the siRNA β-cat condition, which clearly showed a concentration of cell migration toward the cavity region (Fig. S8). The results revealed that siRNA β-cat transfection exhibited a significantly higher velocity compared to the other groups, succeeded by siRNA ITGA5B1 transfection and Y-27632 treatment [Fig. 2(c)]. Nonetheless, the migration speed exhibited a significant decrease in their ability to migrate toward the microcavity under siRNA β-cat transfection. Notably, analysis of the migration trajectories demonstrated that cells with siRNA β-cat transfection tended to migrate diffusely, indicating a loss of coordinated migration [Fig. 2(d)]. Conversely, both Y-27632 treatment and siRNA ITGA5B1 transfection showed persistent collaborative migration, similar to the normal condition, with a strong tendency toward the microcavity. To further interpret cell migration patterns under various conditions, we assessed mean square displacement (MSD) analyses. By fitting MSD to a power function with time iterations, the diffusivity of cell migration was quantified via the power coefficient ***α***. The magnitude of ***α*** offers insights into migration modes: ***α*** = 1 indicates unbiased diffusive motion or random migration, ***α*** < 1 signifies hindered sub-diffusive migration, and ***α*** > 1 suggests directed or persistent migration. Our results indicated that the Y-27632 treatment group has higher ***α*,** which is almost equivalent with the control group [Fig. 2(e)]. This result is also corroborated with the finding, which is also depicted in Fig. 2(d), that control cells predominantly exhibited persistent motion, consistent with aligned migration inferred from migration angles. Y-27632-treated cells displayed a migration pattern similar to control, indicating minimal impact of cell contractility on stacking. However, siRNA ITGA5B1 and siRNA β-cat conditions showed marked diffusivity disparities. Corresponding to Fig. 2(b), siRNA ITGA5B1 exhibited early random migration, transitioning to oriented migration later. Lower MSD for siRNA ITGA5B1 indicated increased early-stage migration randomness. Conversely, siRNA β-cat displayed a notable sub-diffusive migration population (>50%), implying hindered later-stage migration. Collectively, these results emphasize the role of cell-ECM and cell–cell adhesion in 3D stacking migration, with each adhesion mode showing unique migration attributes over time.

### Dependence of cellular stacking on integrin α5β1

**FIG. 3.**
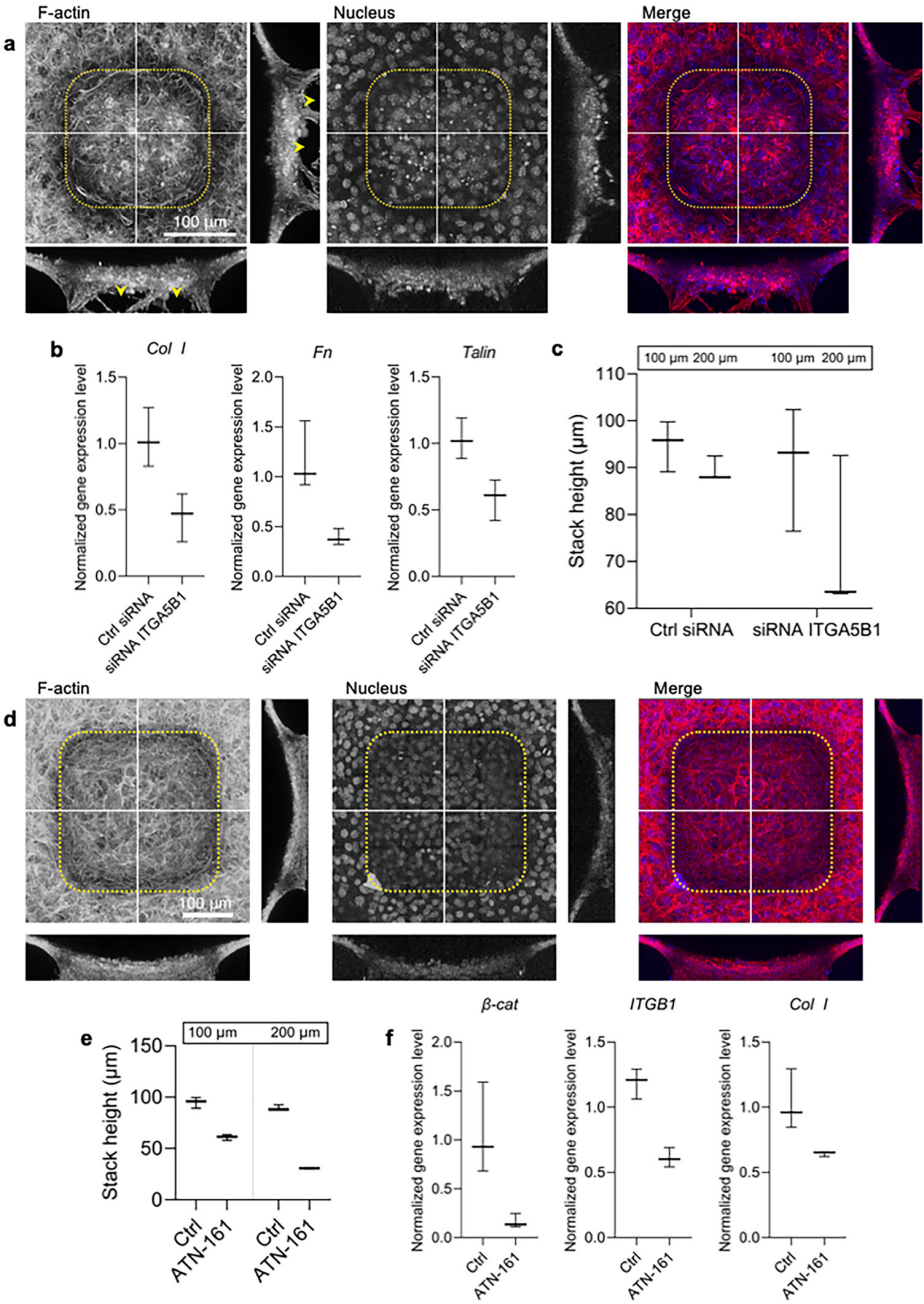
Dependence of stacking cells on integrin α5β1. (a) 3D reconstruction of confocal images showing cells stained for F-actin (red) and nucleus (blue) during siRNA ITGA5B1 transfection. Yellow arrowheads indicate voids in stacking cell formation. Single-channel micrograph have been presented in grayscale to improve contrast and clarity. Scale bar: 100 *μ*m. (b) Gene expression levels of Col I, Fn, and Talin, demonstrating significant downregulation during siRNA ITGA5B1 transfection. Statistical analysis was conducted using Wilcoxon test, comparing the non-treatment group as control, *n =* 3, error bars show standard deviation. (c) Plot of cell heights during siRNA ITGA5B1 transfection. Statistical analysis was conducted using Wilcoxon test, comparing each group with the ctrl siRNA group as the control*, n =* 3, error bars show standard deviation. (d) 3D reconstruction of confocal images showing cells stained for F-actin (red) and nucleus (blue) during ATN-161 treatment. Y–z and x–z sections along white lines are also displayed. Single-channel micrograph have been presented in grayscale to improve contrast and clarity. Scale bar: 100 *μ*m. (e) Quantification plot of cell height during ATN-161 treatment compared to control, demonstrating a significant reduction in cell height with ATN-161 treatment. Statistical analysis was conducted using Wilcoxon test comparing the non-treatment group as control, *n =* 3, error bars show standard deviation. (f) Gene expression levels of β-cat, integrin β1, and Col I, indicating significant downregulation. Statistical analysis was conducted using Wilcoxon test comparing the non-treatment group as control, *n =* 3, error bars show standard deviation.

After verifying the efficacy of the stacking cell formation and the importance of the collective migration toward the microcavity, we proceeded to investigate the role of integrins. Specifically, we examined the contribution of ITGA5B1, known as the fibronectin receptor,^38,39^ to the formation of stacking cells. To assess its significance, we employed siRNA targeting ITGA5B1 and assessed its efficacy during stacking cell formation. We noted that at the final stage, the stacking cells exhibited void spaces, indicated by yellow arrowhead, suggesting an inefficiency in stacking cell formation [Fig. 3(a)]. Additionally, we investigated the response of siRNA ITGA5B1 treatment on related gene expression levels. We then analyzed the gene expression levels of Col I, Fn, and talin, which showed significant downregulation [Fig. 3(b), refer to statistical analysis in the Method section]. These data suggest that void creation, which impedes efficient stacking cell formation, can be attributed to the diminished activity of the ECM and cell–ECM interactions. Conversely, there was no significant variation in the height of the final stacked cells [Fig. 3(c)].

To further validate our findings, we treated the cell culture media with ATN-161, an antagonist of ITGA5B1, after the cells adhered to the microcavity. The results demonstrated a suppression of the total height of stacking cells in the final stage for both L_s_ = 100 and 200 *μ*m sizes [Figs. 3(d) and 3(e), refer to statistical analysis in the Method section], highlighting the importance of ITGA5B1 in stacking cell formation. When examining the gene expression levels of β-cat, integrin β1, and representative extracellular matrix components, such as Col I with ATN-161 treatment, we observed a significant downregulation [Fig. 3(f), refer to statistical analysis in the Method section]. These suggested that ATN-161 treatment disrupts cell–cell adhesion and ECM components, culminating in a diminished cell stature. The formation of the stacking cell possess some characteristics of the spheroid formation, thus, Fn–integrin interaction is also reported to be essential in the structure of the fibroblast spheroid.^40^ Overall, these findings strongly suggest that ITGA5B1 plays a crucial role in effectively filling the microcavities during stacking cell formation.

### Role of cell–cell interactions in layered stromal cell stacking

Building on our understanding of cell–ECM interactions in fibroblast migration, we investigated the role of cell–cell junctions in forming stacked layers through targeted biological assays. Our findings indicate that β-cat and Cx43 are crucial in stromal cell stacking, displaying more significant roles than N-cad. Immunofluorescence staining revealed robust expression of β-cat and Cx43 within the internal regions of cell stacks, while N-cad was predominantly localized at the stack's periphery (Fig. S9). This pattern suggests N-cad facilitates initial cell–cell adhesion, whereas β-cat and Cx43 are integral to the structural integrity and organization of deeper layers. Prior research supports this, showing β-cat's interaction with gap junction proteins such as Cx43 aids in regulating cell differentiation and tissue structuring.^41,42^ The synergy between β-cat's involvement in Wnt signaling and Cx43's role in junction communication is essential for coordinated cellular activity needed for tissue remodeling and ECM production.

**FIG. 4.**
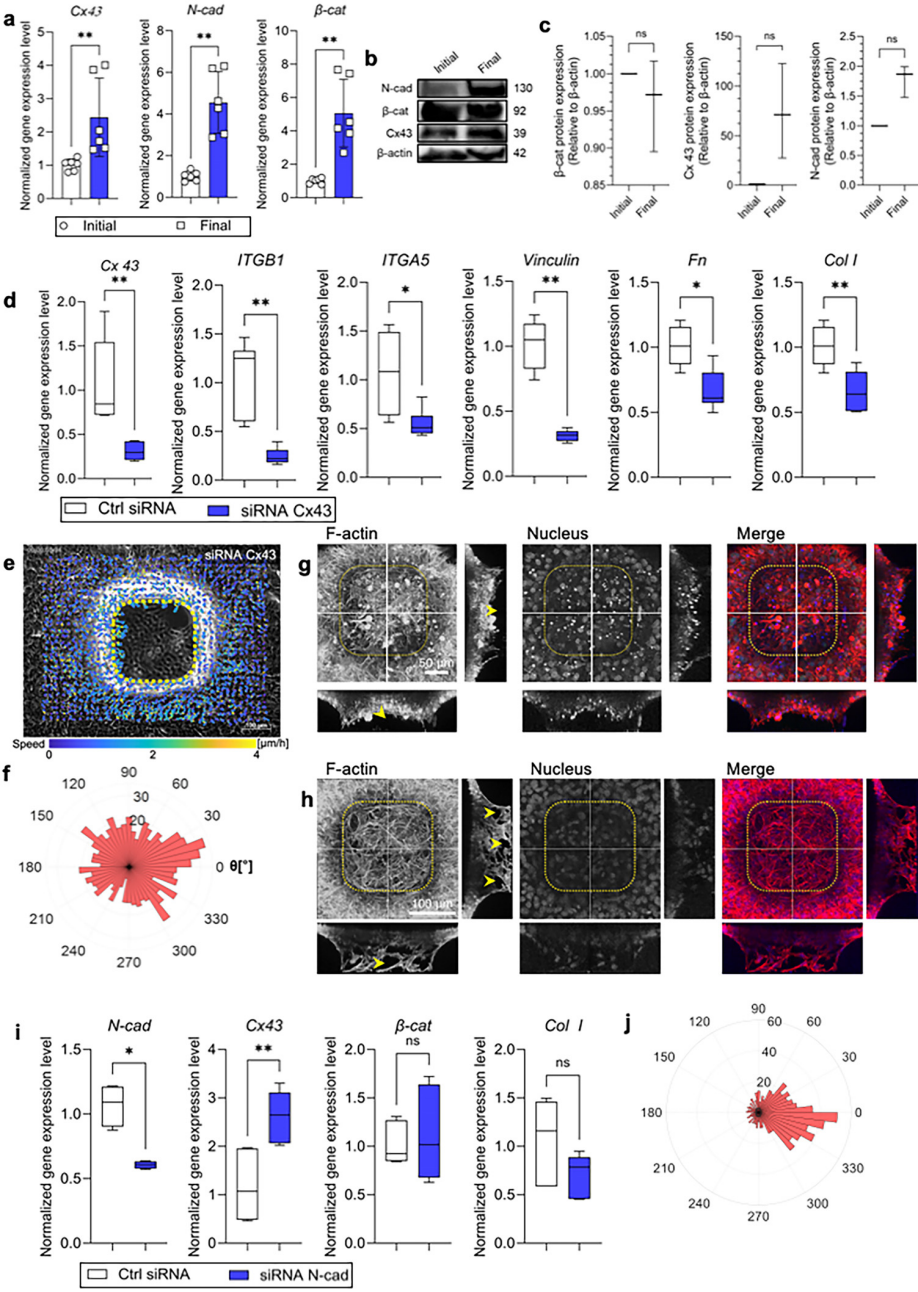
The role of intercellular cell–cell junctions in stacking cell formation. (a) Representative gene expression levels of Connexin 43 (Cx43), N-cad, and (β-cat) comparing initial and final stacking cells. Statistical analysis was conducted using the Kolmogorov–Smirnov test for Cx43 and β-cat while the Mann–Whitney test was used for N-cad, comparing the initial stacking cells as the control. ^**^P < 0.01, *n =* 6, data points represent individual sample, error bars show standard deviation. (b) Band image showing protein quantification results comparing initial and final stacking cells. (c) Plot of protein quantification based on the band images in (b). Statistical analysis was conducted using Wilcoxon test, comparing the initial stacking cells as the control. *n =* 3. (d) Gene expression levels during siRNA Cx43 transfection cells in stacking cell formation. The expression level of siRNA Cx43 is significantly downregulated, along with integrin components (ITGA5 and ITGB1). Focal adhesion (vinculin) is also significantly downregulated, as well as ECM components, such as Fn and Col I. Statistical analysis was conducted using Mann–Whitney test, comparing the ctrl siRNA group as the control. ^**^P < 0.01, ^*^P < 0.05, *n =* 6. (e) Region of interest (ROI) for migration trajectory analysis around the microcavity during siRNA Cx43 transfection. Scale bar: 100 *μ*m. (f) Rose plot showing the migration direction during siRNA Cx43 transfection cells, indicating dispersed migration toward microcavity. (g), (h), 3D reconstruction of the z-projection of confocal images stained for F-actin (red) and nucleus (blue) along the white lines during siRNA Cx43 (g) and siRNA N-cad (h) transfection cells. Yellow arrowheads indicate voids in stacking cell formation, suggesting inefficiency of stacking cell formation. Single-channel micrograph have been presented in grayscale to improve contrast and clarity. Scale bars: 50 *μ*m (g) and 100 *μ*m (h). (i) Gene expression levels during siRNA N-cad transfection cells in stacking cell formation. The results indicate a significant downregulation of N-cad gene expression, while there is an upregulation of Cx43 gene expression and no changes in ECM components, such as Fn and Col I. Statistical analysis was conducted using the Mann–Whitney test, comparing the ctrl siRNA group as the control. ^**^P < 0.01, ^*^P < 0.05, *n* = 6. (j) Rose plot showing the migration direction during siRNA N-cad transfection cells, indicating persistence migration toward microcavity.

Gene expression and protein quantification analyses emphasized a significant increase in cell–cell junction components during the final stacking phases, with the notable exception of protein quantification of β-cat, which remained consistent (Figs. 4(a)–4(c), and S10, refer to statistical analysis in the Method section). Probing further into the functionality of these cell–cell interaction proteins, we introduced siRNAs targeting each protein. Initially, we assessed the efficacy of the siRNA Cx43 and siRNA N-cad and observed downregulated gene expression levels for these targeted genes (Fig. S11, refer to statistical analysis in the Method section). The siRNA experiments targeting Cx43 led to a marked reduction in integrin β1, α5, vinculin, Fn, and Col I levels [Fig. 4(d)], disrupting migration and leading to void formation at the stack's base, as evidenced by Gap 27 inhibition (Fig. S12). This disruption highlighted Cx43's critical role in forming cohesive cell stacks [Figs. 4(e)−(g)].

Conversely, N-cad knockdown had a moderate impact on stack structure, suggesting that while it is vital for initial adhesion, it does not influence the later stages of cell stack formation as significantly as β-cat or Cx43. β-cat and Cx43, therefore, appear more crucial in maintaining cellular communication and architectural organization within the stacks. Our results affirm the hypothesis that β-cat enhances gap junction functionality through its interaction with Cx43, facilitating cell adhesion, migration, and ECM organization—key processes for effective ECM component arrangement and overall tissue stability essential for wound healing.^41,42^

Furthermore, transfection of siRNA targeting N-cad resulted in void formation within the stacking cells, indicating also the inefficient closure [Fig. 4(h)]. Upon conducting real-time PCR analysis, we observed downregulation of N-cad gene expression level, while surprisingly noting upregulation of Cx43, with no gene expression level change in β-cat and Col I [Fig. 4(i)]. Migration trajectory analyses presented a migration modus operandi reminiscent of siRNA ITGA5B1 responses [Fig. 4(j)]. These results indicate that N-cad may plays a role in stacked layers, although it does not significantly affect changes in gene expression levels. Moreover, when we observed the cell morphology during siRNA β-cat, we also noted the void formation, indicated with the yellow arrowheads and significance reduction of the cell height (Fig. S13, refer to statistical analysis in the Method section). These again indicated the significance of β-cat in contributing to the effective stacking cell formation. Contrarily to the scattered migration pattern of fibroblasts on planar substrates,^22,43^ here we showed that fibroblast cells were able to form abundant intercellular junctions. As shown, siRNA-mediated knockdown of Cx43, N-cad, and β-cat resulted in inefficient stacked layers with the void formation, which is due to dispersed cell movement toward microcavity. Depletion of Cx43 through the treatment of Gap 27 indicated that cells were unable to form cell stacking due to the void formation. In essence, our data amplify the pivotal role of intercellular junctions in seamless microcavity filling.

For further analysis, we also observed the role of focal adhesions. We observed an increase in gene expression levels of vinculin and paxillin upon the formation at the final stacking phase [Figs. S14(a) and S14(b), refer to statistical analysis in the Method section). This indicated that vinculin and paxillin (direct binding to vinculin^44^), which also contributed to the cell–cell junction, are abundant in the final stack, thus when cell–cell junctions are inhibited with siRNAs, it leads to inefficient cell stacking. Additionally, our examination of focal adhesion kinase (FAK) staining revealed abundant expression at the basal layer of stacking cells [Fig. S14(c)], suggesting that normal stacking cells exhibit higher cell-ECM adhesion at the basal layer, which led to robust and stable stacking cell formation. In summary, our research emphasizes the dependence of cell stacking on cell–cell junctions, with stack stability being augmented by focal adhesions at the foundational layer.

### Independence of ROCK and RhoA in stacking cell formation

**FIG. 5.**
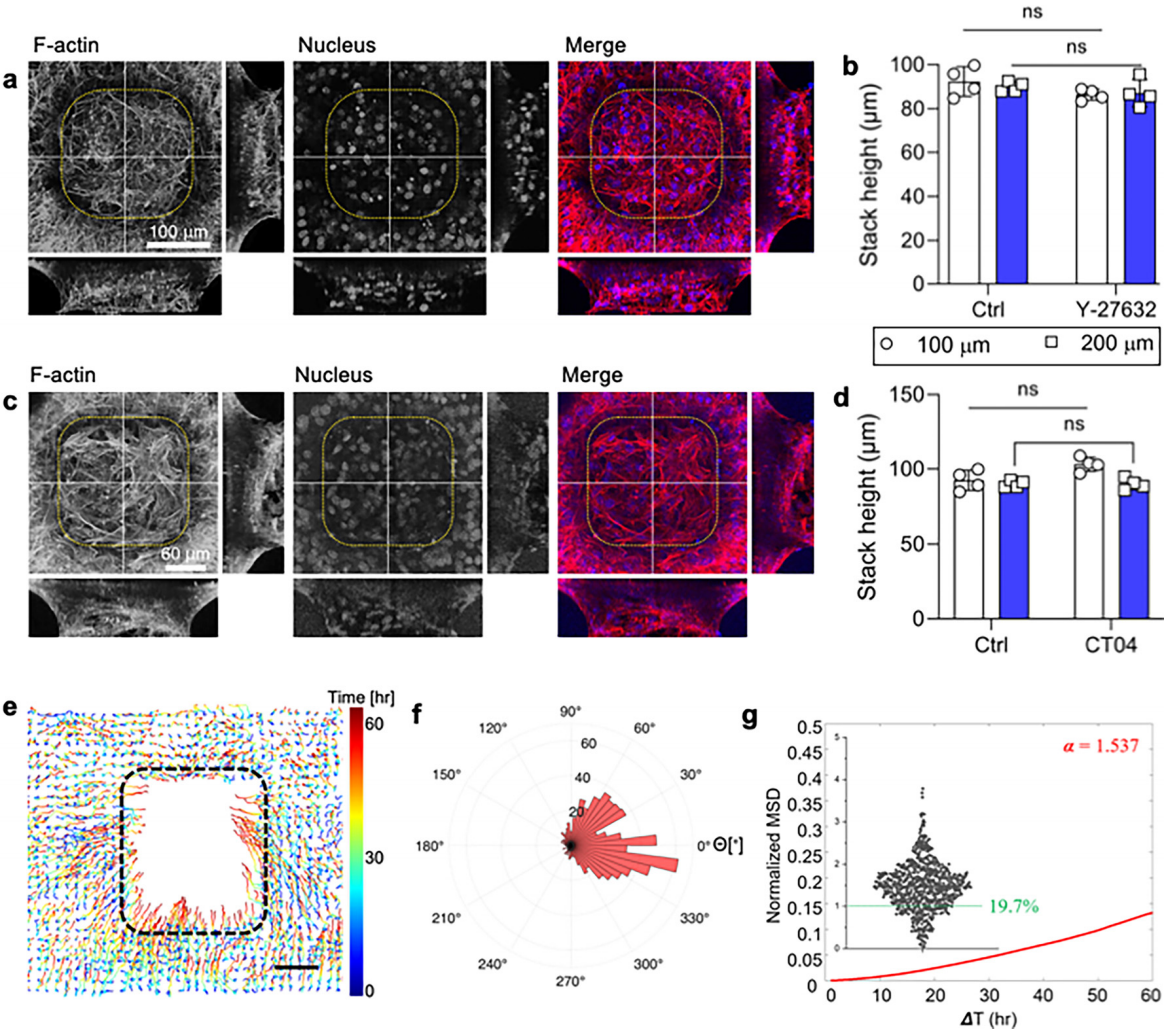
Cytoskeleton contractility does not alter stacking cell formation. (a) 3D reconstruction of confocal images displaying the z-projection, along with corresponding y–z and x–z sections of cells (indicated by white lines). The cells are stained for F-actin (red) and nucleus (blue) during Y-27632 treatment. Single-channel micrographs have been presented in grayscale to improve contrast and clarity. Scale bar: 100 *μ*m. (b) Plot of cell heights comparing control and Y-27632 treatment shows no changes of the stacking cell height. Statistical analysis was conducted using the Kolmogorov–Smirnov test, comparing each group with the ctrl siRNA group as the control*, n = 4,* data points represent individual sample, error bars show standard deviation. (c) 3D reconstruction of confocal images displaying the z-projection, along with corresponding y–z and x–z sections of cells (indicated by white lines). The cells are stained for F-actin (red) and nucleus (blue) during CT-04 treatment. Single-channel micrographs have been presented in grayscale to improve contrast and clarity. Scale bar: 60 *μ*m. (d) Plot of cell heights comparing control conditions and CT-04 treatment indicated there is no effect of the cell height when Rho activity is inhibited. Statistical analysis was conducted using Kolmogorov–Smirnov test, comparing each group with the ctrl siRNA group as the control*, n =* 4, data points represent individual sample, error bars show standard deviation. (e) Analysis of migration trajectories around the microcavity during CT-04 treatment with the progress of time. Scale bar: 100 *μ*m. (f) Rose plot illustrating the migration of the cells along the edge of the microcavity during CT-04 treatment. The result indicated that there is no effect of the cell migration in contributing to the stacking cell formation. (g) MSD analysis result during CT-04 treatment, data points represent individual cell.

Delving deeper, we aimed to elucidate the influence of Rho-associated kinase (ROCK) and RhoA in the stacking cell process. To begin, we allowed the cells to adhere to the substrate and subsequently treated the media with Y-27632, an inhibitor of Rho-associated kinase (ROCK). The results revealed that after 72 h, the cells reached the final stage of stacked layers, which is similar to the ctrl group (untreated) [Fig. 5(a), Movies S10 and S11]. Additionally, the measurements of the stacking cell height indicated similarity to those of normal stacked layers, approximately 82–88 μm for a L_s_ = 100 *μ*m [Fig. 5(b)]. Attributing this observation, we posited that the consistent migration of cells toward the microcavity and an elevated MSD might be causal factors. From these data, we inferred that ROCK plays a non-critical role in the stacking cell generation. To further support these data, we treated the cell culture media with CT-04, an inhibitor of RhoA. The results corroborated with the findings of the Y-27632 treatment, demonstrating that stacking cell formation was unaffected with the morphology of the stacking cell formation [Fig. 5(c)]. Additionally, there is no significant difference of the cell height compared to the control [Fig. 5(d)]. We also analyzed the trajectories of the cell migration toward microcavity using PIV and found that cells are persistently migrated toward the microcavity [Figs. 5(e) and 5(f)]. Furthermore, we also noted that MSD is relatively higher than siRNA ITGA5B1 and β-cat, the integrin and cell–cell adhesions, even though slightly lower than the control group [Figs. 2(e) and 5(g)]. Throughout this collective migration process, cells crawl along the steep curvature toward the microcavity. However, the ROCK/RhoA pathway, the major regulating mechanism of the cell migration, is confirmed to have less influence on the stacking migration. This may be due to the fact that stacked layers are mainly driven by the cell–ECM interaction and cell–cell adhesion. Collecting all these together, the data indicate that both ROCK and RhoA, often integral to cellular migration dynamics, seem non-essential in the context of stacking cell development. This implies that stacking cell formation has different migration behaviors from the mesenchymal or epithelial cell migration behavior in a confined microchannel, where cell behavior is affected and varied by the differential contractility.^45–47^

## CONCLUSIONS

In this study, we have delineated the intricate dynamics of stromal cell collective migration within a confined 3D microcavity environment. Stromal cells were successfully able to form a stacking within a confined microcavity ranging from L_s_ = 100–400 *μ*m, with a cell thickness of approximately 108 *μ*m in the case of a 100 *μ*m microcavity. Interestingly, the final stage of this stacking cell formation exhibits a heightened level of the ECM components, including as Fn and Col I. The analysis of the pattern of the collective cell migration using PIV confirmed that normal stacking cell formation has the behavior of coordinated migration from the reservoir toward the microcavity, which is also corroborated with the migration pattern of Y-27632 treatment. Our findings highlight that cell-substrate adhesion via integrin α5β1 and intercellular interaction, such as Cx43, N-cad, and β-cat, play pivotal roles in mediating collective cell migration. The effectiveness of the stacked layers relies heavily on the persistence and dispersal migration of the cells toward the microcavity. However, we found that the RhoA/ROCK signaling pathway is not affected the efficiency of stacking cell formation in the microcavity. With these insights, we anticipate that our findings will wield transformative potential in the realm of tissue engineering, especially in elucidating wound healing nuances during granulation tissue formation stages.

## METHODS

### 3D microcavity sample preparation

The pattern of the master mold was designed using AutoCAD software, which was subsequently transferred for mask fabrication. The patterned mask was printed and applied to a stainless-steel substrate (which become the master mold), after which the exposed surfaces were chemically etched. To enhance the peel-off process, the fabricated stainless-steel mold was coated with a 10 nm thick layer of polytetrafluoroethylene (PTFE) using deep reaction-ion etching, reducing adhesion between PDMS and the stainless-steel mold. The 3D microcavities were synthesized by thoroughly mixing a curing agent and base from the PDMS kit (Sylgard 184, Dow Chemical) at a ratio of 1:10 (curing agent to base). The mixed PDMS solution was then poured into the stainless-steel mold and subjected to heat treatment for rapid curing at 150 °C for 10 min, following the specifications provided by Dow Corning Inc. Once cured, the microcavity sample was peeled off from the mold to serve as the 3D microcavity substrate.

### PDA coating

3-Hydroxytyramine hydrochloride (dopamine hydrochloride, catalog no. H8502, Sigma-Aldrich Korea) was dissolved in 10 mM Tris buffer (pH 8.5) to 2 mg/mL as a polydopamine (PDA) solution. To enhance the wettability of the PDMS-based microcavity samples and improve cell adhesion, a surface treatment using dip-coating in a PDA solution was employed. In brief, the PDMS microcavity sample was initially rinsed with de-ionized water and then immersed in the PDA solution for a duration of 2 min. Following this, the sample was removed and subjected to two subsequent immersions in de-ionized water to eliminate any excess PDA solution on the PDMS-based microcavity sample. The sample was dried by blowing nitrogen gas and stored in a desiccator for subsequent cell culture in this study. While oxygen (O_2_) plasma is a common method to enhance the hydrophilicity of PDMS surfaces, this approach requires an additional layer of ECM proteins for adequate cell adhesion. In contrast, PDA coating provides dual benefits. It not only increases surface hydrophilicity but also inherently promotes cell attachment due to the presence of adhesive molecules like DOPA and lysine. These molecules enable PDA to form robust covalent and non-covalent bonds with the PDMS substrate, offering a more streamlined and efficient method to create a cell-adhesive environment.

### Cell culture

NIH3T3 mouse fibroblast (ATCC CRL-1658) cells, human dermal fibroblast cells (ATCC PCS-201-012), and HaCaT cells were maintained in DMEM supplemented with 10% of fetal bovine serum (FBS) and antibiotics (100 U mL^−1^ penicillin and 100 *μ*g mL^−1^ streptomycin; Gibco, Grand Island, NY, USA). Normal culture conditions were maintained at 37 °C and 5% CO_2_ in a humidified incubator. Throughout all the experiments, the initial cell seeding is 100 000 cells/cm^2^. Cell culture is performed either in a confocal dish (100 350, SPL life science) for immunofluorescence staining analysis or 6-well plate tissue culture plate for real-time PCR or western blot.

### Immunofluorescence staining

To perform immunofluorescence staining, first, cells were washed with PBS 1x (70011-044, Gibco) three times and then fixed with 4% paraformaldehyde (P0117CD-500ML, Bylabs, Korea) at room temperature for 30 min. Next, cells were rinsed with PBS 1x three times. The cells were permeabilized with 0.2% triton X-100 in PBS 1x for 10 min and subsequently washed with PBS 1x three times then blocked with 3% bovine serum albumin (BSA) in PBS 1x for 1 h. The samples were then incubated with primary antibody in 1% BSA at room temperature (RT). List of primary antibodies are as follows: anti-β-cat antibody (ab16051, Abcam), anti-Col I antibody (ab34710, Abcam), anti-fibronectin antibody (sc-8422, Santa Cruz), anti-Cx43 antibody (3512S, Cell Signaling Technology), and anti-N-cad antibody (4061S, Cell Signaling Technology). The samples were then rinsed with PBS 1x and followed by 1 h incubation with rhodamine phalloidin (R415, Thermo Fisher Scientific), 4′, 6-diamidino-2-phenylindole (DAPI, Thermo Fisher Scientific), and secondary antibodies, such as goat anti-mouse Alexa Fluor 488 secondary antibody (A11001, Invitrogen) and goat anti-rabbit Alexa Fluor 488 secondary antibody (A11008, Invitrogen), in 1% BSA at RT. Next, the sample was then washed with PBS 1x three times and was made ready for image acquisition using a laser scanning confocal microscope (Zeiss LSM 700; Carl Zeiss Micro-Imaging GmbH, Germany). For F-actin and nucleus, images were prepared as described above except without primary and secondary antibodies. The images were processed using Imaris 8.0.1, briefly, the scanned images were processed by section view, 3D, or sliced view for image extraction. Section view displays three images at one shot, x–y (right top), x–z (right bottom), and y–z (left) sections. For 3D morphology, section view is a favorable option to display how the morphology of the taken images viewed in all sections. 3D view displays all the layer of the taken image in one content. 3D view is favorable for regenerating the image into a motion movie as microcavity. All the motion movies were made at 250 frames per second. Slice view of the image will extract all layer-by-layer images.

### Cell counting

The number of cells in each microcavity is counted using the Imaris software. Briefly, the volume of the microcavity is selected to prevent the inclusion of the number of cells on the reservoir. Next, at the scene section spots are added to detect the nucleus of the cells and then skip automatic creation is chosen and edited manually. Under the main spot tab we chose 8 as the radius size for the cell counting.

### ATN-161, Y-27632, and CT-04 treatments

ATN-161 (SML2079-25MG, Sigma-Aldrich) is treated to the cell culture media with the concentration of 1 *μ*M/mL throughout this study. Y-27632 (Y0503, Sigma) is treated to the cell culture media with the concentration of 25 *μ*M after the adhesion time, 4 h prior to the cell seeding on the microcavity. CT04 (Cytoskeleton Inc) treatment is done according to the protocol from the company. Briefly, cells were seeded on the microcavity and after 4 hrs of the adhesion time the cell culture media is treated with CT04 with the concentration of 2 *μ*g/mL for 2 h.

### Live-cell imaging

After cells adhere on the microcavity, the samples were transferred to the live cell imaging incubator and maintained at the standard culture condition of 37 °C and 5% CO_2_ in humidified atmosphere. The videos were acquired by taking the images every 1 h using Zeiss live cell imaging confocal with the objective lens of 10x and the z-interval of 10 *μ*m (Zeiss live cell confocal, Observer. Z1, Germany).

### PIV analysis

PIV analysis is performed after the acquisition of the images from live cell imaging with the duration of 3 days. To measure the cellular velocity, particle image velocimetry (PIV) analysis was conducted by using the Image Velocimetry Tool for MATLAB (version: 1.43) software. We used double-pass PIV by the fast Fourier transform (FFT) window deformation algorithm with a first window size of 64 × 64 pixels and a second window size of 32 × 32 pixels. Velocity vectors were distributed with a 16-pixel interval (20 *μ*m), similar to the body length of a cell. The trajectory of cells was analyzed from the velocity fields. The first location of each trajectory was allocated by dividing the whole region with 32 × 43 windows. The next location was updated by adding the displacement of each window, which was calculated from the PIV data (Fig. S7).

### siRNA transfection

The transfection of the siRNAs was performed according to the protocol from the company (Bioneer Korea). Briefly, 24 h prior to the transfection, normal cell culture media was changed with the absence of antibiotics. Next, predesign siRNA such as siRNA integrin α5 (siRNA ID: 109700-1; Bioneer, Korea), siRNA integrin β1 (siRNA ID: 16412-1; Bioneer, Korea), siRNA β-cat (12387-2, Bioneer, Korea), siRNA Cx43 (siRNA ID: 14609–2, Bioneer Korea), N-cad (siRNA ID: 12558-2, Bioneer, Korea), and negative control of siRNA (AccuTarget™ Negative Control siRNA, Bioneer, Korea) were used and mixed with the Lipofectamine RNAiMAX Transfection Reagent (Invitrogen) as the carrier. The transfection is done for 24 hrs with the concentration of 50 nM for all siRNAs.

### Real-time-PCR

For quantitative polymerase chain reaction (qPCR) analysis, cells were seeded on a specialized designed microcavity (Fig. S5) and the total mRNA was extracted from the cells using 1 ml Trizol^®^ RNA isolation reagents (Invitrogen). The lysis is done for all the cells including on the reservoir and stacking cells in the microcavity. Concentration and purity of the samples were quantified using NanoDrop ND-1000 spectrophotometer (Thermo Fisher Scientific). cDNA synthesis was then performed by adding 4 *μ*L of Super-Script^®^ VILO^TM^ into each sample in MicroAmp^®^ Optical 8-Tube Strip (Applied Biosystems). The synthesis is done at 45 °C for 60 min and RTase inactivation at 95 °C for 5 min. Next, the synthesized cDNA (1 *μ*L), 10 pmol of each reverse and forward primer in 2 *μ*L Diethyl pyrocarbonate (DEPC)-treated water, 10 *μ*L of SYBR Green real-time PCR mix (RR420A; Takara), 0.4 *μ*L of ROX reference dye II, and 6.6 *μ*L DEPC were mixed in PCR reaction tubes (Applied Biosystems) to a total volume of 20 *μ*L. The reaction reagents were then placed in a real-time cycler (7500 Real-Time PCR System; Life Technologies™) and relative gene expression level was calculated by 2^-ΔΔCt^ method and normalized with GAPDH as the housekeeping gene. Target genes and their primer sequences were listed in Table I (supplementary materials).

### Immunoblotting assay

Cells were lysed using RIPA buffer (89 900, Thermo scientific) with the supplement of 1X protease inhibitor cocktail EDTA-free (87 785, Thermo scientific). The samples were boiled with 1X SDS-PAGE sample loading buffer (Biosesang, Korea). The samples were loaded for electrophoresis using Mini-PROTEAN TGX Gels (4 561 023, Bio-Rad). Transferring is done using polyvinylidene fluoride (PVDF) membrane. The samples were blocked with 5% skim milk and incubated with following primary antibodies: N-cad (ab98952, Abcam), β-cat (ab16051, Abcam), Cx43 (3512S, Cell Signaling), ITGA5 (ab150361, Abcam), ITGB1 (PA5-29606, Invitrogen), and β-actin (C4, Life science) in 4 °C overnight. The samples were washed three times in Tris Buffered Saline with Tween (TBST) 1x and then the samples were incubated in the HRP-conjugated secondary antibodies, for anti-mouse (31430, Invitrogen) and anti-rabbit (VJ313046, Thermo Scientific) for 1 h at room temperature. Next, the blot in TBST is rinsed three times and then the chemiluminescent substrate (34 580, Thermo Scientific) is applied to the blot. iBright CL1500 (Thermo Fisher Scientific) was used to capture the chemiluminescent signal. All quantified data were normalized with β-actin.

### Statistical analysis

For statistical analysis, we used GraphPad Prism 5 for all the analysis. In this study, we applied Kolmogorov–Smirnov test for Figs. 1(c), 1(d), 4(a), 5(b), and 5(d) as these data follow normal distribution. For those which do not meet normal distribution, we used nonparametric statistical Wilcoxon test for Figs. 1(h), 3(b), 3(c), 3(e), 3(f), 4(c), S3(b), S3(c), S4(f), S4(g), S5(b), S11, S14(a), and S14(b). Additionally, we also utilized the Mann–Whitney test for Figs. 4(d), 4(i), S6(a), S6(c), S6(e), and S13(b). In addition, the statistical analysis for the cell migration result is analyzed using the Kruskal–Wallis test (SPSS, IBM). Statistical significance is marked as ^*^P < 0.05, ^**^P < 0.01, ^***^P < 0.001, and ^****^P < 0.0001). Furthermore, the figures [Fig. 1(h), 3(b), 3(e), 3(f), 4(c), S3, S4(f), S4(g), S5(b), S6(c), S11, S13(b), S14(a), and S14(b)] have significant differences using SPSS; however, due to the small sample size, statistical significances are not indicated.

## SUPPLEMENTARY MATERIAL

See the supplementary material for the characterization of the 3D microcavity; 3D reconstruction images of F-actin and nucleus of cells on L_s_ = 200, 300, and 400 *μ*m; 3D reconstruction images of F-actin (red) and nucleus (blue) in hDFB cells along white lines on L_s_ = 100 *μ*m; inability of HaCaT cells to form stacking structures on 3D microcavities; characterization of the microcavity substrate for real-time PCR and immunoblotting assays; confirmation of the downregulation of integrin α5 and integrin β1 gene expression levels during siRNA ITGA5B1 treatment; methodology for tracking cell trajectories by updating the virtual positions of cells from the interpolated velocity fields; velocity component changes during the stacking up migration according to the inhibition of ROCK, ITGA5B1, and β-cat; three-dimensional reconstruction of the z-projection of confocal images stained for F-actin (red), nucleus (blue), and Cx43, β-cat, and N-cad (green) at the final stage of stacking formation; uncut membrane from the western blot result; optimization of siRNA targeting Cx43 and N-cad assessed via real-time PCR; effect of gap 27 treatment on cell stacking formation; response of the stacking cells during siRNA β-cat; role of FA in stacking formation; gene expression level of the vinculin and Paxillin; and list of primers and sequences used in this study.

## Data Availability

The data that support the findings of this study are available from the corresponding authors upon reasonable request.
